# Statistics of the instantaneous interaural parameters for dichotic tones in diotic noise (N_0_S_*ψ*_)

**DOI:** 10.3389/fnins.2022.1022308

**Published:** 2022-11-08

**Authors:** Jörg Encke, Mathias Dietz

**Affiliations:** ^1^Physiology and Modeling of Auditory Perception, Department of Medical Physics and Acoustics, University of Oldenburg, Oldenburg, Germany; ^2^Cluster of Excellence “Hearing4all”, University of Oldenburg, Oldenburg, Germany

**Keywords:** sound localization, probability density function, interaural level difference, interaural phase difference, tone in noise detection, binaural unmasking

## Abstract

Stimuli consisting of an interaurally phase-shifted tone in diotic noise—often referred to as *N*_0_*S*_*ψ*_—are commonly used to study binaural hearing. As a consequence of mixing diotic noise with a dichotic tone, this type of stimulus contains random fluctuations in both interaural phase- and level-difference. We report the joint probability density functions of the two interaural differences as a function of amplitude and interaural phase of the tone. Furthermore, a second joint probability density function for interaural phase differences and the instantaneous cross-power is derived. The closed-form expression can be used in future studies of binaural unmasking first to obtain the interaural statistics and then study more directly the relation between those statistics and binaural tone detection.

## 1. Introduction

Tone in noise detection thresholds improve when the interaural configuration of tone and noise differ compared to the diotic case. A rich literature reports on the influence of virtually every parameter of acoustic stimuli on this binaural unmasking (see, e.g., Culling and Lavandier, 2021, for a review). Amongst these parameters, the phase difference *ψ* introduced between the target tones of the two ear signals is fundamental and was explored already in the first study of dichotic tone in noise detection by Hirsh (1948). Such a signal is commonly referred to as N_0_S_*ψ*_ where the subscripts indicate the interaural phase difference (IPD) of the noise (N) or signal (S). The difference between the detection threshold for the purely diotic N_0_S_0_ and the N_0_S_*ψ*_ signal is referred to as the binaural masking level difference (BMLD) and is largest for the case where *ψ* = π (Hirsh, 1948).

**Figure 1:**
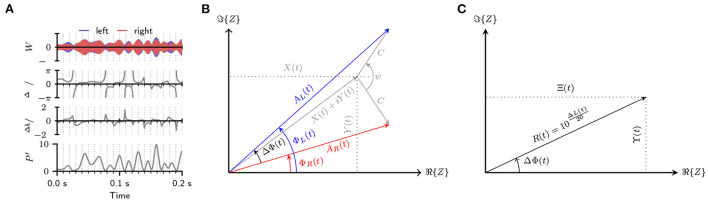
**(A)** Visualization of the random fluctuations in IPD ΔΦ(*t*) and ILD Δ*L*(*t*) and *P*′(*t*) due to mixing an antiphasic 500 Hz tone with a 500 Hz wide band of diotic noise (SNR = −10 dB). **(B)** Signal model used to derive the PDFs for an N_0_S_*ψ*_ stimulus. The graphic shows the Complex-plane representation of the basebands of the left and right ear signal: $Z_{L}\left(t\right)=A_{L}\left(t\right){\text{e}}^{i\Phi_{L}\left(t\right)}$ (blue), and $Z_{R}\left(t\right)=A_{R}\left(t\right){\text{e}}^{i\Phi_{R}\left(t\right)}$ (red). The left-ear-baseband is constructed by adding a “tone”-vector with length *C* and angle +*ψ*/2 to the noise baseband *X*(*t*)+*iY*(*t*). The right-ear-signal is constructed by adding a “tone”-vector with an angle of −*ψ*/2 to the same baseband. The instantaneous IPD ΔΦ(*t*) of the N_0_S_*ψ*_ signal equal the difference between Φ_*R*_ and Φ_*L*_. **(C)** Complex-plane representation of the interaural-baseband *Z*_1_(*t*) = Ξ(*t*) + *i*ϒ(*t*) which is gained by dividing the left-ears-baseband by the right-ears-baseband. The absolute value of the baseband equals the interaural amplitude ratio *R* while the phase equals the interaural phase difference ΔΦ.

Adding a dichotic *S*_*ψ*_ tone to diotic *N*_0_ noise reduces the correlation between the left and right signals but also introduces random fluctuations of the interaural phase and level differences (IPD, ILD) (visualized in Figure 1A). The interaural correlation decreases with the tone level, so binaural unmasking and incoherence detection are often treated synonymously (Durlach et al., 1986). However, especially for narrowband noise, the value of interaural correlation itself was found to be an insufficient predictor for decorrelation detection performance. Instead, detection performance correlated with the amount of IPD and ILD fluctuations as measured by the standard deviation (Goupell and Hartmann, 2006). Similarly, other studies reported the performance in detecting the tone within an N_0_S_*ψ*_ stimulus to vary considerably depending on the individual noise token. This token to token variability was best accounted for by models that did consider the amount of instantaneous fluctuations in IPD and ILD (Davidson et al., 2009).

Therefore, accounting for binaural tone-in-noise sensitivity can be subdivided into two components: First, the signal-based analysis of how stimulus design parameters such as *ψ* or the SNR influence the interaural cue statistics. In the second step, binaural sensitivity can then be studied more directly by relating it to the interaural cues. Only relatively few studies, however, have previously treated these statistics. The probability density function (PDF) underlying the statistical distribution of IPDs in (partly) decorrelated noise has been derived in the frame of optical interferometry (Just and Bamler, 1994). Henning (1973) derived the PDF for IPDs in the special case of N_0_S_π_ and using a very similar approach for the same stimulus condition, Zurek (1991) additionally derived marginal PDFs for ILDs. Other studies also seemed to have worked on stimuli where the tone IPD did not equal π, but this work seemed to have remained unpublished (Levitt and Lundry, 1966). The present study closes this gap by deriving a closed form expression for the joint PDF of IPDs and ILDs in the general case of a *N*_0_*S*_*ψ*_ stimulus. From this distribution, the marginal PDFs can also be calculated using numerical integration. These PDFs are especially useful when considering narrowband noises that remain relatively unaffected by the bandpass properties of the auditory periphery. Statistics at the stimulus level should thus well describe statistics of the binaural parameters at the level of binaural integration.

Suppose fluctuations of the IPD are indeed a cue used to detect the tone in an N_0_S_*ψ*_ stimulus. In that case, the stimulus energy at which these fluctuations occurred might also affect performance. A larger IPD occurring during low-energy stimulus sections can be expected to have less impact than the same IPD occurring at high stimulus energy. Information about the stimulus energy in both ears is captured by the product of the left and right ear stimulus envelope, also called the instantaneous cross-power *P*′(*t*). Furthermore, the cross-power plays an essential role in defining the interaural coherence of a stimulus (Encke and Dietz, 2022). Consequently, this study derives the joint PDF for *p*′(*t*) and IPD.

## 2. Deriving the probability density functions

The following section will derive the two joint PDF. A computational-notebook that can be used to reproduce these derivations in the computer algebra system sympy (Meurer et al., 2017) can be found as Supplementary material.

If *N*(*t*) is a Gaussian noise process with a mean value of zero, the process can be represented using its in-phase and quadrature components *X*(*t*) and *Y*(*t*):

(1) $$\begin{array}{l} N\left(t\right)=X\left(t\right)\cos\left(\omega_{0}t\right)-Y\left(t\right)sin\left(\omega_{0}t\right), \end{array}$$

where *X*(*t*) and *Y*(*t*) are orthogonal noise processes with the same variance and mean as *N*(*t*). The reference frequency ω_0_ is not of relevance for the derivation and can thus be chosen freely. For computational convenience, ω_0_ is set to equal the frequency of the tone *S*(*t*) which is added with the amplitude *C* and phase *ψ*:

(2) $$\begin{array}{l} S\left(t\right)=C\sin\left(\omega_{0}t+\psi \right) \end{array}$$

The resulting signal *W*(*t*) = *N*(*t*) + *S*(*t*) then equals:

(3) $$\begin{array}{l} W\left(t\right)=\left[X\left(t\right)+C\cos\left(\psi \right)\right]\cos\left(\omega_{0}t\right)\\ \;-\left[Y\left(t\right)+C\sin\left(\psi \right)\right]\sin\left(\omega_{0}t\right). \end{array}$$

When dealing with instantaneous phase and amplitude values, it is beneficial to instead work with the analytic representation *W*_*a*_(*t*) of the signal:

(4) $$\begin{array}{l} W_{a}\left(t\right)=\left\{\left[X\left(t\right)+C\cos\left(\psi \right)\right]+i\left[Y\left(t\right)+C\sin\left(\psi \right)\right]\right\}{\text{e}}^{i\omega_{0}t}, \end{array}$$

where $i=\sqrt{-1}$ is the imaginary unit. The first term of this expression (enclosed in curly brackets) can be interpreted as an amplitude and phase modulator of the harmonic oscillation ${\text{e}}^{i\omega_{0}t}$. This combined modulator will be referred to as the signals complex baseband *Z*(*t*)

(5) $$\begin{array}{l} Z\left(t\right)=\left[X\left(t\right)+C\cos\left(\psi \right)\right]+i\left[Y\left(t\right)+C\sin\left(\psi \right)\right]=A\left(t\right){\text{e}}^{i\Phi \left(t\right)}, \end{array}$$

where *A*(*t*), Φ(*t*) are the instantaneous amplitude and phase of the baseband. In the case of the N_0_S_*ψ*_ stimulus, a tone with phase *ψ*/2 is added to the noise in the left-ear signal while the phase of the tone in the right-ear signal is −*ψ*/2 resulting in the two basebands:

(6) $$\begin{array}{l} Z_{\text{L}}\left(t\right)=\left[X\left(t\right)+C\cos\psi /2\right]+i\left[Y\left(t\right)+C\sin\psi /2\right]=A_{\text{L}}\left(t\right){\text{e}}^{i\Phi_{\text{L}}\left(t\right)} \end{array}$$

(7) $$\begin{array}{l} Z_{\text{R}}\left(t\right)=\left[X\left(t\right)+C\cos-\psi /2\right]+i\left[Y\left(t\right)+C\sin-\psi /2\right]\\ \;=A_{\text{R}}\left(t\right){\text{e}}^{i\Phi_{\text{R}}\left(t\right)}. \end{array}$$

A vector model of the basebands *Z*_R_ and *Z*_L_ is shown in Figure 1B where the individual components are visualized as vectors in the complex plane.

Based on these two basebands, PDFs for the interaural parameters will be derived using two separate approaches. In the first approach, the baseband of the left-ear signal *Z*_*L*_(*t*) is divided by the baseband of the right-ear signal *Z*_*R*_(*t*) resulting in the interaural baseband *Z*_1_(*t*):

(8) $$\begin{array}{l} Z_{1}\left(t\right)=\frac{Z_{R}\left(t\right)}{Z_{L}\left(t\right)}=\frac{A_{R}\left(t\right)}{A_{L}\left(t\right)}{\text{e}}^{i\left[\Phi_{R}\left(t\right)-\Phi_{L}\left(t\right)\right]}\\ \;=R\left(t\right){\text{e}}^{i\Delta \Phi \left(t\right)}, \end{array}$$

where ΔΦ(*t*) and *R*(*t*) are the instantaneous IPDs and the interaural amplitudes ratios (IARs), respectively. Instantaneous ILDs can then be calculated as: Δ*L*(*t*) = 20 log_10_ *R*(*t*). In the second approach, the PDF for IPDs and the product of the left and right-ear envelope (cross power) *p*′ are derived by multiplying *Z*_L_(*t*) with the complex conjugate of *Z*_R_(*t*) resulting in

(9) $$\begin{array}{l} Z_{2}\left(t\right)=Z_{R}\left(t\right)Z_{L}^{*}\left(t\right)=A_{R}\left(t\right)A_{L}\left(t\right){\text{e}}^{i\left[\Phi_{R}\left(t\right)-\Phi_{L}\left(t\right)\right]}\\ \;=P^{\prime }\left(t\right){\text{e}}^{i\Delta \Phi \left(t\right)}. \end{array}$$

The process of deriving the PDFs from Equation (8) and Equation (9) follows the exact same rationale so that the process will only be detailed for Equation (8). Results for the second approach will then be stated without further detail.

For the interaural baseband, *Z*_L_ and *Z*_R_ as resulting from Equations (6) and (7) are inserted into Equation (8) resulting in:

(10) $$\begin{array}{l} Z_{1}\left(t\right)=\frac{\left[X\left(t\right)+C\cos\left(\psi /2\right)\right]+i\left[C\sin\left(\psi /2\right)+Y\left(t\right)\right]}{\left[X\left(t\right)+C\cos\left(-\psi /2\right)\right]+i\left[C\sin\left(-\psi /2\right)+Y\left(t\right)\right]}\\ \;=\Xi \left(t\right)+iϒ\left(t\right) \end{array}$$

where Ξ(*t*) and ϒ(*t*) are the in-phase and quadrature components of the baseband *Z*_1_(*t*). They can be derived from Equation (10) as:

(11) $$\begin{array}{l} \Xi \left(t\right)=\frac{Y^{2}\left(t\right)+{\left[C\cos\left(\psi /2\right)+X\left(t\right)\right]}^{2}-C^{2}\sin^{2}\left(\psi /2\right)}{{\left[C\sin\left(\psi /2\right)-Y\left(t\right)\right]}^{2}+{\left[C\cos\left(\psi /2\right)+X\left(t\right)\right]}^{2}} \end{array}$$

(12) $$\begin{array}{l} ϒ\left(t\right)=\frac{2C\left[C\cos\left(\psi /2\right)+X\left(t\right)\right]\sin\left(\psi /2\right)}{{\left[C\sin\left(\psi /2\right)-Y\left(t\right)\right]}^{2}+{\left[C\cos\left(\psi /2\right)+X\left(t\right)\right]}^{2}}. \end{array}$$

Figure 1B visualizes the resulting baseband in the complex plane. From this visualization, it can be seen that the instantaneous IPDs and IARs can be calculated as the argument: ΔΦ(*t*) = arg{*Z*_1_(*t*)} = arctan2(ϒ(*t*), Ξ(*t*)) and modulus $R\left(t\right)=|Z_{1}\left(t\right)|=\sqrt{ϒ{\left(t\right)}^{2}+\Xi {\left(t\right)}^{2}}$ of the baseband. Here, arctan2 is the two-argument arctangent that returns the angle in the Euclidean plane.

Both Random Processes *R*(*t*) and ΔΦ(*t*) are functions of *X*(*t*) and *Y*(*t*) which are uncorrelated Gaussian noise processes with the variance σ^2^. The joint PDF *f*_*X, Y*_(*x, y*) of *X*(*t*) and *Y*(*t*) is thus that of a bivariate Gaussian distribution:

(13) $$\begin{array}{l} f_{X,Y}\left(x,y\right)=\frac{1}{2\pi \sigma^{2}}{\text{e}}^{-\frac{1}{2\sigma^{2}}\left(x^{2}+y^{2}\right)}, \end{array}$$

where

(14) $$\begin{array}{l} \overset{\infty }{\underset{-\infty }{\iint }}f_{X,Y}\left(x,y\right)\text{d}x\text{d}y=1. \end{array}$$

Here and in all future equations, lower-case variables will be used to refer to the individual instances generated by a given noise process. *x* and *y* are thus two instances generated by the noise processes *X*(*t*) and *Y*(*t*) and ξ, υ are generated by Ξ(*t*) and ϒ(*t*).

Probability density functions for Ξ(*t*) and ϒ(*t*) can be gained by applying a coordinate transformation to Equation (13). For this, Equations (11) and (12) are rearranged to calculate *x* and *y* given the values of ξ and υ:

(15) $$\begin{array}{l} x\left(\xi ,\upsilon \right)=C\left[\frac{2\upsilon \sin\left(\psi /2\right)}{\upsilon^{2}+{\left(\xi -1\right)}^{2}}-\cos\left(\psi /2\right)\right],\\ y\left(\xi ,\upsilon \right)=\frac{C\left(\upsilon^{2}+\xi^{2}-1\right)\sin\left(\psi /2\right)}{\upsilon^{2}+\xi^{2}-2\xi +1}. \end{array}$$

These expressions allow us to derive the Jacobian determinant |*J*(*x, y*)|. The Jacobian is then used to apply a coordinate transformation from d*x* and d*y* to dξ and dυ:

(16) $$\begin{array}{l} \text{d}x\text{ d}y=|J\left(x,y\right)|\text{d}\xi \text{ d}\upsilon =\frac{4C^{2}\sin^{2}\left(\psi /2\right)}{{\left[\upsilon^{2}+{\left(\xi -1\right)}^{2}\right]}^{2}}\text{d}\xi \text{ d}\upsilon . \end{array}$$

Applying the transformations in Equations (15) and (16) to change the variables of Equation (13) results in:

(17) $$\begin{array}{l} f_{\Xi ,ϒ}\left(\xi ,\upsilon \right)\\ \;=\frac{2C^{2}\sin^{2}\left(\psi /2\right)}{\pi \sigma^{2}{\left[\upsilon^{2}+{\left(\xi -1\right)}^{2}\right]}^{2}}{\text{e}}^{-\frac{C^{2}\left[\upsilon^{2}-2\upsilon \sin\left(\psi \right)+\xi^{2}-2\xi \cos\left(\psi \right)+1\right]}{2\sigma^{2}\left[\upsilon^{2}+{\left(\xi -1\right)}^{2}\right]}}. \end{array}$$

Which is the joint PDF for the two random processes Ξ(*t*) and ϒ(*t*). To gain the joint PDF *f*_*R*, ΔΦ_(*r*, Δφ), Equation (17) is transformed from rectangular to polar coordinates (see Figure 1C). This is achieved by using the transforms: ξ = *r* cos Δφ, υ = *r* sin Δφ, dξ dυ = *r* d*r* dΔφ resulting in:

(18) $$\begin{array}{l} f_{R,\Delta \Phi }\left(r,\Delta \varphi \right)=\frac{C^{2}2r\sin^{2}\left(\psi /2\right)}{\sigma^{2}\pi h{\left(0\right)}^{2}}{\text{e}}^{-\frac{C^{2}h\left(\psi \right)}{\sigma^{2}2h\left(0\right)}}, \end{array}$$

where *h*(*ψ*) = *r*^2^ − 2*r* cos(Δφ − *ψ*) + 1 and *r* ∈ [0, ∞], Δφ ∈ [−π, π].

**Figure 2:**
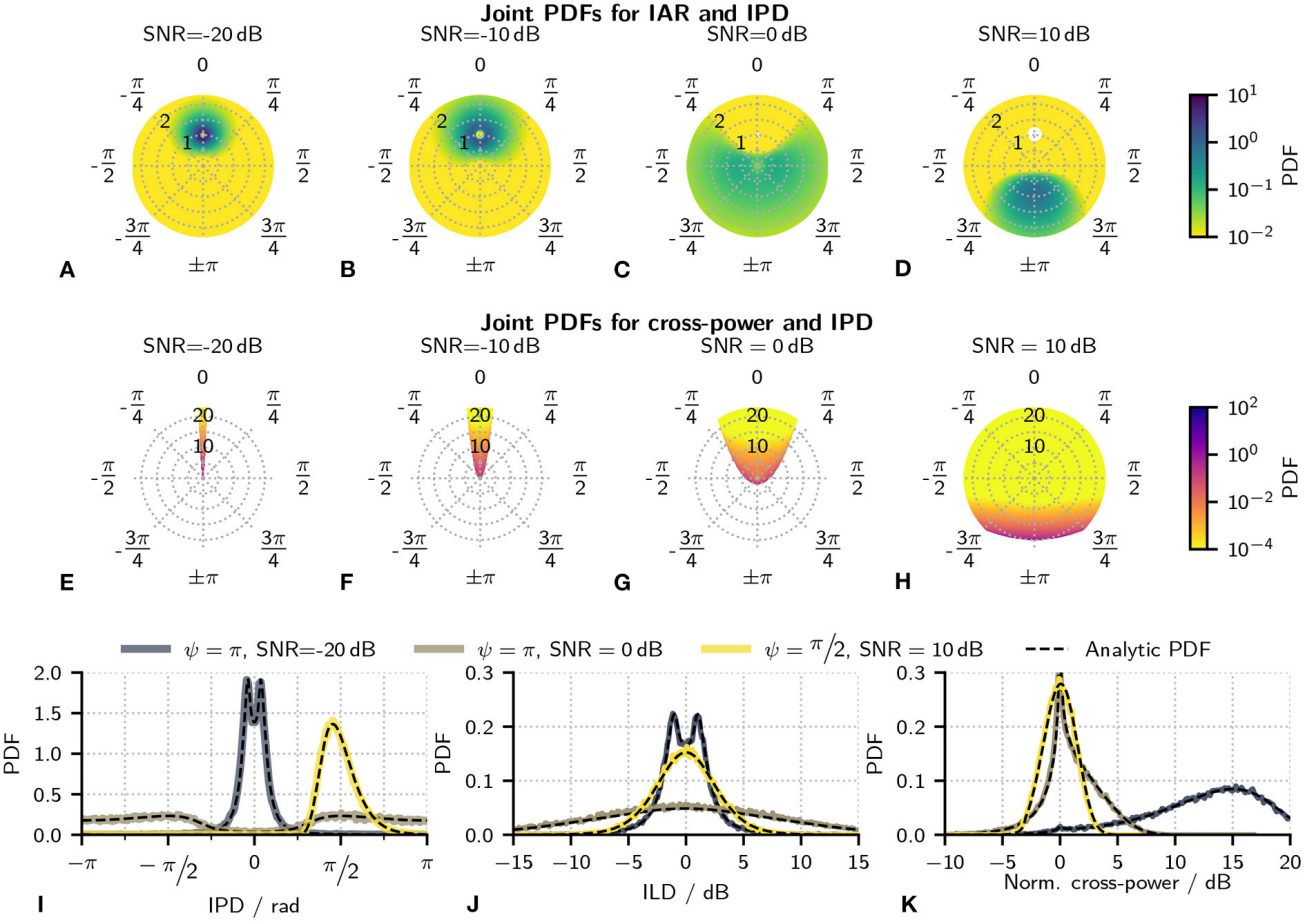
**(A–D)** Some examples of the joint PDF of IAR and IPD given in Equation (18). All plots show results for a tone-IPD of π with the SNR increasing from left to right. Angles in the polar plot are the IPDs, while the radial variable is the IAR. Colors indicate the probability density. A logarithmically-scaled colormap was used due to the large dynamic range of the PDF. White areas located at an IAR = 1 and IPD = 0 for 0 and 10 dB indicate a probability density of 0. **(E–H)** Joint PDF for cross-power and IPDs given in Equations (20). Results are shown for the same parameters as in **(A–D)**. As in the first row of plots, angles indicate the IPD and color the probability density. The radial variable, however, is the cross-power. These PDFs were calculated for a noise variance of σ^2^ = 1. A logarithmically-scaled colormap was used due to the large dynamic range of the PDF. White areas indicate undefined combinations of cross-power and IPD as defined by Equation (23). **(I–K)** Evaluation of the analytical results by comparing the derived marginal PDFs with numerically estimated PDFs. In all cases, black, dashed lines indicate analytical results gained from Equations (24)–(27). Colored lines indicate results that were instead numerically estimated from waveforms. Panel **(I)** shows marginal PDFS for IPDs ΔΦ, **(J)** for ILDs Δ*L* and k) for the cross-power *P*′.

This equation can be interpreted as the distribution of all possible values of the interaural baseband $z_{1}=r{\text{e}}^{i\Delta \varphi }$ and thus the distribution of all possible combinations of IPDs Δφ and IARs *r*. It is also apparent from Equation (18) that equal ratios of *C*^2^/*σ*^2^ result in the same PDF so that PDFs will be referenced using the signal to noise ratio SNR = *C*^2^/2*σ*^2^ instead of σ^2^ and *C*. Some examples of these functions are shown in Figures 2A–D. Deriving the joint PDF of Δφ and ILD Δ*l* instead of IAR *r* is easily done by using transforms *r* = 10^Δl/20^ and *dr* = r/20 ln(10)*d*Δ*l*.

(19) $$\begin{array}{l} f_{\Delta L,\Delta \Phi }\left(\Delta l,\Delta \varphi \right)=\frac{C^{2}10^{\Delta l/20}\ln\left(10\right)\sin^{2}\left(\psi /2\right)}{\sigma^{2}\pi h{\left(0\right)}^{2}}{\text{e}}^{-\frac{C^{2}h\left(\psi \right)}{\sigma^{2}2h\left(0\right)}}. \end{array}$$

To derive the joint PDF of ΔΦ(*t*) and *P*′(*t*), the process detailed above is repeated based on the interaural baseband *Z*_2_(*t*) as defined in Equation (9) resulting in the PDF:

(20) $$\begin{array}{l} f_{P^{\prime },\Delta \Phi }\left(p^{\prime },\Delta \varphi \right)=\frac{e^{-\frac{C^{2}}{2\sigma^{2}}-\frac{p^{\prime }\left[\cos\left(\Delta \varphi \right)-\cos\left(\Delta \varphi -\psi \right)\right]}{2\sigma^{2}\left[\cos\left(\psi \right)-1\right]}}p^{\prime }}{2\pi \sigma^{2}\sqrt{g}} \end{array}$$

where *g* is given by:

(21) $$\begin{array}{l} g=2C^{2}\sin^{2}\left(\psi /2\right)\left[2p^{\prime }\cos\left(\Delta \varphi \right)-C^{2}\left(\cos\left(\psi \right)-1\right)\right]\\ \;-{p{\;}^{\prime }}^{2}\sin^{2}\left(\Delta \varphi \right). \end{array}$$

and the range of values is defined by:

(22) $$\begin{array}{l} p^{\prime }\in \left[0,\hat{p^{\prime }}\left(\Delta \varphi \right)\right],\;\Delta \varphi \in \left[-\hat{\Delta \varphi }\left(p^{\prime }\right),+\hat{\Delta \varphi }\left(p^{\prime }\right)\right], \end{array}$$

where

(23) $$\begin{array}{l} \hat{p^{\prime }}\left(\Delta \varphi \right)=C^{2}\frac{\cos\left(\psi \right)-1}{\cos\left(\Delta \varphi \right)-1}. \end{array}$$

The function $\hat{\Delta \varphi }\left(p^{\prime }\right)$ can be gained by solving Equation (23) for Δφ.

Similar to Equation (19) which defined the distribution of all possible values of Δφ and *r*, this function can be interpreted as the distribution of all possible combinations of Δφ and *p*′. However, the range of these combinations is limited by Equation (23) so that large areas of the exemplary PDFs shown Figures 2E–H are undefined. This limitation will be treated further in the discussion.

The marginal PDFs of the IAR *R*, the IPD ΔΦ and the cross-power *P*′ can be calculated from the two joint PDFs defined in Equations (19) and (20) by integrating over the other variable.

(24) $$\begin{array}{l} f_{\Delta \Phi }\left(\Delta \varphi \right)=\int_{0}^{\infty }f_{R,\Delta \Phi }\left(r,\Delta \varphi \right)\text{d}r\\ \;=\int_{0}^{\hat{p^{\prime }}\left(\Delta \varphi \right)}f_{P^{\prime },\Delta \Phi }\left(p^{\prime },\Delta \varphi \right)\text{d}p^{\prime } \end{array}$$

(25) $$\begin{array}{l} f_{\text{R}}\left(r\right)=\int_{-\pi }^{\pi }f_{R,\Delta \Phi }\left(r,\Delta \varphi \right)\text{d}\Delta \varphi \end{array}$$

(26) $$\begin{array}{l} f_{P^{\prime }}\left(p^{\prime }\right)=\int_{-\hat{\Delta \varphi }\left(p^{\prime }\right)}^{\hat{\Delta \varphi }\left(p^{\prime }\right)}f_{P^{\prime },\Delta \Phi }\left(p^{\prime },\Delta \varphi \right)\text{d}\Delta \varphi \end{array}$$

(27) $$\begin{array}{l} f_{\Delta L}\left(\Delta l\right)=\int_{-\pi }^{\pi }f_{\Delta L,\Delta \Phi }\left(\Delta L,\Delta \varphi \right)\text{d}\Delta \varphi . \end{array}$$

As previously discussed, the PDFs of Δφ and Δ*l* (and thus *r*) only depend on the SNR and not on the absolute stimulus power. The cross-power *P*′, however, is the product of the left and right stimulus envelope and must thus also depend on stimulus power. For this reason, PDFs for *P*′ will always be shown normalized by *C*^2^ so that PDFs only depend on the SNR and are independent of overall stimulus power.

No closed-form solution for Equations (24)–(27) could be found so that numeric integration was used to evaluate them (QUADPACK algorithms QAGS/QAGI; Piessens et al., 1983). Figures 2I–K show some examples of the PDF of ΔΦ, Δ*L*, *P*′ and verifies the results by comparing Equations (24)–(25) to PDFs that were numerically estimated from signal waveforms.

## 3. Discussion

Figures 2A–D show joint PDFs for IAR and IPD calculated at a tone-IPD of *ψ* = π and different SNRs. Without any tone, this distribution would equal a delta distribution with infinite probability density at an IPD of zero and an IAR of 1. At low SNRs (Figures 2A,B), the antiphasic tone has only a small influence on the noise resulting in probability densities that are still tightly clustered around the IPD of 0 and an IAR of 1. With increasing amplitude of the tone and thus increasing SNR, this clustering becomes less pronounced (Figures 2B,C). When the tone starts to dominate the stimulus, the probability density becomes highest around the tone-IPD of π (Figures 2C,D). At large SNRs, the PDF would converge toward a delta distribution at the tone-IPD of π and an IAR of 1. Figures 2E–H shows joint PDFs for cross-power and IPD at the same conditions as used in Figures 2A–D. Without the antiphasic tone, the stimulus density would be concentrated on a single line at zero IPD. Also, the signal is diotic so that the cross-power equals the stimulus power so that the cross-power distribution would equal that of the squared envelope. At low SNRs (Figures 2E,F), the addition of the tone starts to introduce IPD fluctuations thus widening the joint PDF. A large area of these joint PDFs are, however, undefined. These undefined areas are determined by Equation (23) and become intuitive when studying the signal model shown in Figure 1B. At low tone amplitudes *C*, it is only possible to gain large IPDs at moments where the envelope of the noise and thus *x* + *iy* are small. This also result in a small cross-power $p^{\prime }=a_{L}\times a_{R}$. With increasing *C*, large IPDs can then also appear at increasingly large values of *p*′. This is seen in Figure 2G and especially Figure 2H.

**Figure 3:**
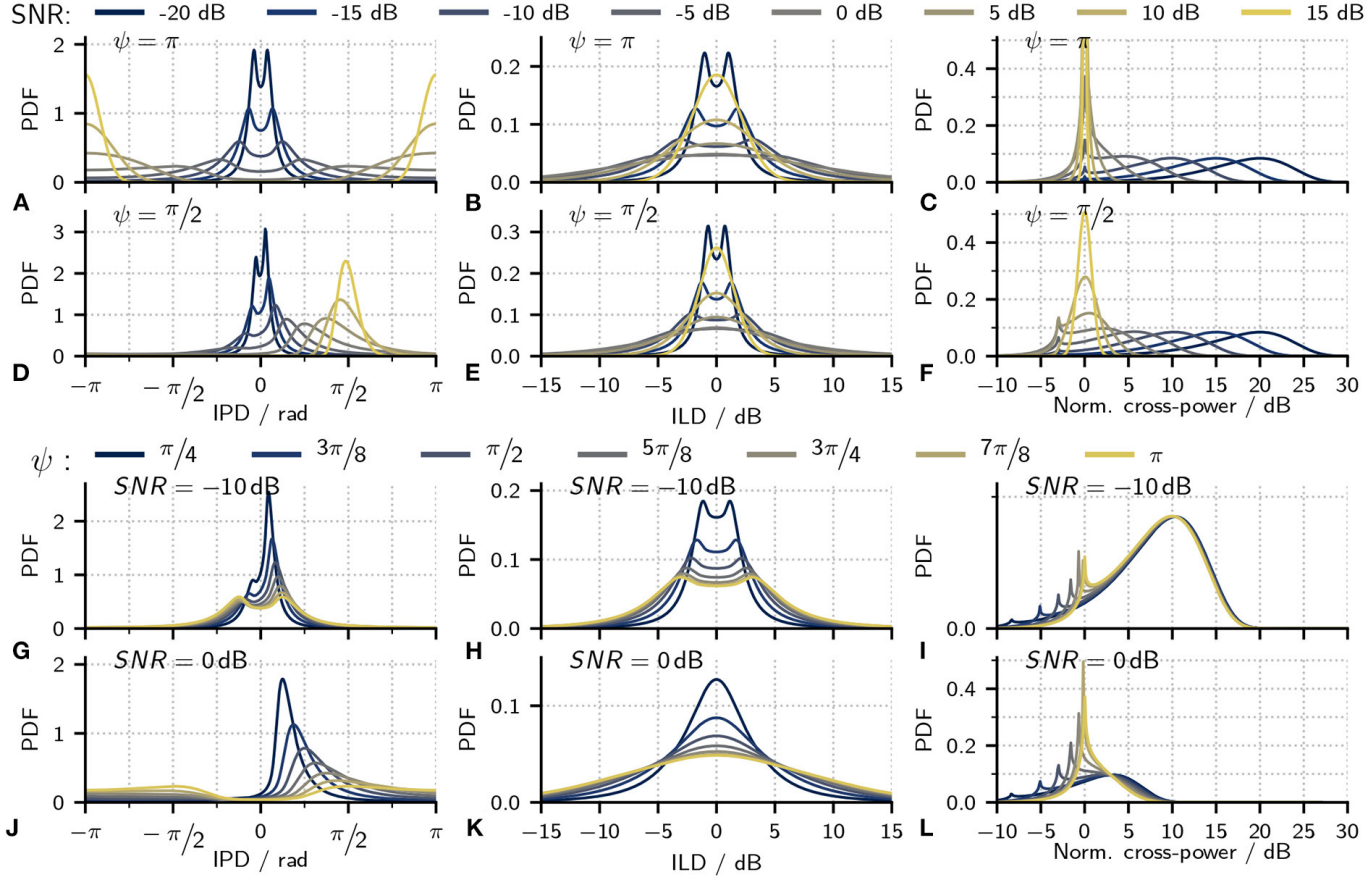
Exemplary marginal PDFs for IPDs (first column), ILDs (second column), and the cross-power (third column). For better visualization, the cross-power values were normalized with the squared tone amplitude so that the x-axis shows $10\log_{10}\left(p^{\prime }/C^{2}\right)$. **(A–F)** PDFs calculated for two fixed signal phases *ψ* = π (top-row) and *ψ* = π/2 (bottom row). Different colors indicate results at different SNRs. **(G–L)** PDFs calculated for two fixed SNRS: −10 dB (top-row) and 0 dB (bottom row). Different colors indicate results at different signal phases *ψ*.

While joint PDFs are the main contribution of this study, they are hard to visualize and, consequently, difficult to discuss in detail. Instead, the following section discusses marginal PDFs for IPDs, cross-power, and ILDs as a function of different stimulus properties. These PDFs lack information about the interaction between the individual metrics, such as IPD and cross-power or ILD. However, they do convey the impact of different metrics more intuitively. Figures 3A,D show examples of the marginal IPD PDFs for *ψ* = π and *ψ* = π/2 while varying the SNR. The instantaneous IPD Δφ can be interpreted as a result of the mixture of zero IPD due to the diotic noise and the IPD *ψ* of the tone. The weighting of the two IPDs is determined by the noise's instantaneous power relative to the tone's power. Thus, at large negative SNRs where the stimulus is dominated by noise, IPD PDFs show a mean value close to zero and only little variance. With increasing SNR, the IPDs are increasingly influenced by the tone-IPD so that the distributions mean moves toward *ψ* and variance increases. At larger positive SNRs, where the noise power is small compared to the tone, the IPDs are dominated by the tone-IPD *ψ* so that the variance decreases again. In the two extreme cases where the SNR would either be −∞ or +∞, the signal consists of only the noise or the tone so that neither IPD nor ILD fluctuates—both PDFs are then δ-distributions. For the IPD, this distribution is either be located at zero (SNR=−∞) or at *ψ* (SNR=+∞) while the ILD distribution is always centered at 0 dB. Figures 3B,E show ILD PDFs for the same parameters as used for the IPD PDFs in Figures 3A,D. Instantaneous ILDs Δ*l*, are a direct result of the relative energy of the instantaneous noise and the tone. As a result, ILD PDFs exhibit the same change of variance as discussed for the IPDs, low variance at both high or low SNRs where the stimulus is either dominated by the tone or noise and an increase of variance at intermediate SNRs. Figures 3C,F show distributions for the remaining parameter *P*′ plotted in decibels relative to the squared amplitude of the tone. For large SNRs, the signal is dominated by the tone, p′/C^2^ is thus narrowly distributed around 0 dB. With decreasing SNR, the noise power increases relative to *C*^2^ so that the peak of the distribution shifts toward larger values of *p*′/*C*^2^ with the overall shape of the distribution remaining largely unchanged.

**Figure 4:**
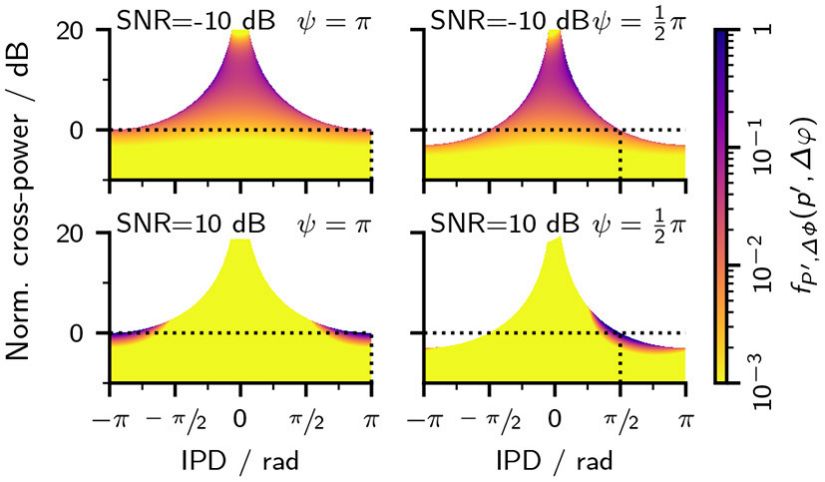
Joint probability functions of the cross-power *P*′ and IPD as defined in Equation (20). For better comparison, the y-axis was normalized with the squared tone amplitude so that the y-axis shows $10\log_{10}\left(p^{\prime }/C^{2}\right)$. The top row shows PDFs at an SNR of −10 dB, while the bottom row shows PDFs at an SNR of 10 dB. Columns show Each panel shows a PDF at different SNRs and Tone-IPDs *ψ*. The horizontal dashed black lines indicate the location where *p*′ = *C*^2^ so that the normalized cross-power is 0 dB. The vertical black lines indicate where the IPD matches the tone-phase Δφ = *ψ*. Note that the color map is logarithmically-scaled and that changes in the scale were limited to values between 1 and 10^−3^.

Figures 3G–L additionally show IPD, ILD, and *P*′ PDFs for cases where the SNR was fixed while varying *ψ*. From the vector summation shown in Figure 1B, it is intuitive that, at the same tone amplitude *C*, a smaller value of *ψ* also results in smaller IPDs. As a direct consequence, IPD and ILD PDFs also show less variance for smaller values of *ψ*. The PDFs for *P*′, however, are largely uninfluenced by *ψ*—with the notable exception of a sharp peak located at *p*′/*C*^2^ = sin^2^(*ψ*/2). This peak is a consequence of Equation (23), which limits the possible combinations of IPDs and *P*′. To better understand the origin of this peak, Figure 4 shows joint PDFs of IPD and *P*′. Notably, the probabilities are heavily clustered close to the limit defined by Equation (23). The low slope of the limiting ${\hat{p}}^{\prime }$ function toward ±π in combination with the accumulation of probability density along this limit results in the observed peak in the cross-power PDFs. From Equation (23) follows that ${\hat{p}}^{\prime }\left(\Delta \varphi =\pm \pi \right)=C^{2}\sin^{2}\left(\psi /2\right)$ which is the location of the peaks in Figures 3I,L.

All PDFs derived above show discontinuities for Δφ ∈ {0, ±π} for which the probability densities approach zero. Or, in other words, a N_0_S_*ψ*_ stimulus will never contain IPDs that are exactly zero or π. Both discontinuities can be understood when keeping in mind that the IPD is defined by Δφ = arctan2(υ, ξ). Which can only result in a value of 0 or ±π if υ = 0. This is only the case when *x* = −*C* cos (*ψ*/2). As the probability of *x* to take this exact value approaches zero, the joint PDFs will also approach zero. For further discussion of the PDFs, however, this discontinuity was not shown explicitly in the plots above as its implication in practice is limited.

Furthermore, the PDFs derived in this study are independent of noise spectrum and bandwidth. They are thus valid for any Gaussian noise with zero mean. Further, the tone frequency does not need to be located within the noise spectrum. However, with auditory processing, especially peripheral filtering, the spectrum will influence the effective SNR at the level of binaural interaction and, thus, the PDFs of the encoded binaural cues. In these cases, PDFs will be determined by the effective SNR of the stimulus as processed, meaning after considering the bandpass properties of the auditory periphery. While all PDFs were derived for the diotic noise case N_0_S_*ψ*_, they can easily be generalized to cases where an additional phase delay *ψ*_2_ is applied to the whole stimulus. Such a signal could then be referred to as (*N*_0_*S*_*ψ*_)_*ψ*_2__ and would result in identical IPD distributions as in the *N*_0_*S*_*ψ*_ case but shifted by *ψ*_2_ with ILD and *P*′ distributions remaining unchanged.

### 3.1. Quantifying IPD and ILD variability

Multiple studies have used models making use of the variability of IPDs, ILDs, or a combination of the two, as a detection cue for tone in noise experiments (e.g., Davidson et al., 2009; Dietz et al., 2021; Encke and Dietz, 2022; Eurich et al., 2022) or for decorrelation detection (Goupell and Hartmann, 2007). Based on the derived PDFs, the following section will thus discuss different measures for the amount of IPD and ILD fluctuation for the special case of N_0_S_π_.

**Figure 5:**
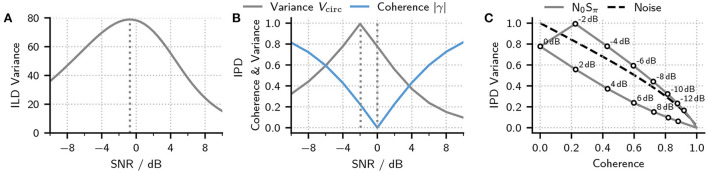
**(A)** Variance of ILDs in an N_0_S_π_ signal calculated at different SNRs. The dashed line marks the maximum of the function **(B)** Circular IPD variance in an N_0_S_π_ signal calculated at different SNRs (blue line) and the matching interaural coherence (gray line). Dotted lines indicate the location of the maximum in variance and minimum in coherence. **(C)** Circular IPD variance as a function of stimulus coherence for an N_0_S_π_ stimulus (gray line and symbols) as well as (partly) decorrelated noise (dashed black line) (Just and Bamler, 1994). Symbols and labels indicate SNRs resulting in a given combination of coherence and variance.

The amount of ILD fluctuations can be quantified by calculating the variance *V* of the underlying distribution defined as:

(28) $$\begin{array}{l} V=<\Delta L{\left(t\right)}^{2}>=\overset{\pi }{\underset{-\pi }{\int }}\int_{-\infty }^{\infty }\Delta l^{2}f_{\Delta L,\Delta \Phi }\text{d}\Delta l\text{ d}\Delta \varphi , \end{array}$$

where the angular brackets symbolize the ensemble average. The resulting variance as a function of SNR is shown in Figure 5A. As expected from the plots in Figure 3, ILD variance first increases with SNR until reaching its maximum around an SNR of −0.73 dB from where the variance decreases as the tone starts to dominate the stimulus.

Most previous studies relied on the regular variance (or standard deviation $\sqrt{V}$) as defined in Equation (28) when quantifying IPD variance (Goupell and Hartmann, 2007; Davidson et al., 2009). This approach makes sense at low SNRs where IPDs are narrowly distributed around 0. At higher SNRs, however, the distribution starts to move toward a mean value of π, and calculating the regular variance is of little significance. An alternative and better-suited metric for quantifying the IPD variability is the circular variance *V*_circ_ (Fisher, 1993) defined as:

(29) $$\begin{array}{l} V_{\text{circ}}=1-|\langle {\text{e}}^{i\Delta \Phi \left(t\right)}\rangle |=1-|\overset{\pi }{\underset{-\pi }{\int }}\int_{0}^{{\hat{p}}^{\prime }\left(\Delta \varphi \right)}{\text{e}}^{i\Delta \varphi }f_{P^{\prime },\Delta \Phi }\text{d}p^{\prime }\text{d}\Delta \varphi |, \end{array}$$

where the angular brackets symbolize the ensemble average, *V*_circ_ can take values between 0 and 1 with a value of 0 indicating no IPD fluctuations. In contrast, a value of 1 indicates a wide distribution of IPDs (but not necessarily a uniform distribution). The gray line shows the circular variance as a function of SNR in Figure 5B. Like the ILD variance, IPD variance increases with increasing SNR until reaching its maximum around an SNR of −1.93 dB from where the variance starts to decrease.

A second and alternative metric for quantifying the amount of IPD fluctuations has recently been shown to directly account for the detection performance in a variety of tone in noise tasks: The interaural coherence^1^ |γ| (Encke and Dietz, 2022; Eurich et al., 2022). The interaural coherence is defined as the modulus of the complex-valued correlation coefficient and can be calculated as:

(30) $$\begin{array}{l} |\gamma |=\frac{\left|\langle R_{a}\left(t\right)L_{a}^{*}\left(t\right)\rangle \right|}{<\sqrt{|R_{a}\left(t\right){|}^{2}><|L_{a}\left(t\right){|}^{2}>}}=\frac{\left|\langle P^{\prime }\left(t\right){\text{e}}^{i\Delta \Phi \left(t\right)}\rangle \right|}{<\sqrt{|R_{a}\left(t\right){|}^{2}><|L_{a}\left(t\right){|}^{2}>}}, \end{array}$$

(31) $$\begin{array}{l} =\frac{1}{2\sigma +C^{2}}|\overset{\pi }{\underset{-\pi }{\int }}\int_{0}^{{\hat{p}}^{\prime }\left(\Delta \varphi \right)}p^{\prime }{\text{e}}^{i\Delta \varphi }f_{P^{\prime },\Delta \Phi }\text{d}p^{\prime }\text{d}\Delta \varphi |, \end{array}$$

where *R*_*a*_, *L*_*a*_ are the analytical representation of the left and right ear signals, the asterisk symbolizes the complex conjugate, and σ^2^ and *C* are the variance of the noise and the amplitude of the tone, respectively. Comparing this equation to the definition of *V*_circ_ in Equation (29), shows that the two measures are closely related, with the main difference being that |γ| weights the IPDs by *p*′ before averaging. This weighting requires a normalization achieved by the term before the integrals. In addition to this, the two metrics show inverse behavior. A stimulus with no IPD fluctuations will result in an interaural coherence of |γ| = 1 while the circular variance would be *V* = 0.

An interesting property of |γ| is that any stimulus with a real-valued cross power density spectrum such as N_0_S_π_ also results in a real-valued γ which then equals the interaural (Pearson) correlation. Figure 5B shows the interaural coherence (and thus correlation) as a function of SNR (blue line). As expected from the previous discussions, the coherence decreases with increasing SNR until reaching a coherence of zero at an SNR of 0 dB from where it starts to increase. Surprisingly, however, the minimum in coherence does not match the maximum in IPD or ILD variability. Figure 5C thus shows the same data as in panel b but plotting IPD variance as a function of coherence. The same plot also shows the IPD variance of two partly correlated noise tokens as a function of coherence. From this figure, one can appreciate that, depending on the stimulus, the same coherence can result in different amounts of IPD variance. These differences are caused by the *p*′ weighting of IPDs that is included when calculating |γ| (see Equation 31). two stimuli that share the same IPD PDF but differing *P*′ PDFs would thus show also differ in their coherence.

## 4. Summary

This study aimed to derive the joint PDF for ILDs (IARs) and IPDs as well as IPDs and *P*′. The two functions are given by the Equations (19) and (20). The two equations are a key component for understanding how the SNR and *ψ* influence the magnitude of binaural unmasking when considering IPD and ILD variance as the underlying cue. The approach applied to derive PDFs can further be used as a template for other types of binaural signals. In the future, it will hopefully help to get a better understanding of how different stimulus statistics influence binaural unmasking.

## Data availability statement

The original contributions presented in the study are included in the article/Supplementary material, further inquiries can be directed to the corresponding author/s.

## Author contributions

JE and MD designed the research and wrote the paper. JE conducted the calculations, analyzed the data, and produced the figures. All authors contributed to the article and approved the submitted version.

## Funding

This work was supported by the European Research Council (ERC) under the European Union's Horizon 2020 Research and Innovation Programme grant agreement No. 716800 (ERC Starting Grant to MD).

## Conflict of interest

The authors declare that the research was conducted in the absence of any commercial or financial relationships that could be construed as a potential conflict of interest.

## Publisher's note

All claims expressed in this article are solely those of the authors and do not necessarily represent those of their affiliated organizations, or those of the publisher, the editors and the reviewers. Any product that may be evaluated in this article, or claim that may be made by its manufacturer, is not guaranteed or endorsed by the publisher.

## Footnotes

^1^Note that there are several different definitions of coherence. Our use of coherence as |γ| is a typical time-domain definition (Saleh, 2007). In general signal processing, the coherence function is instead often defined in the frequency domain and calculated as the normalized absolute value of the cross-spectral power density (CSPD) (Shin, 2008). The two definitions are closely related, as the time-domain coherence can also be defined by using a Fourier transform of the CSPD. In binaural research, a third definition exists, where interaural coherence is sometimes used to refer to the maximum of the real-valued cross-correlation function (Blauert, 1983).
