# Supplementary material for: Statistics of the instantaneous interaural parameters for dichotic tones in diotic noise (N0Sψ)
Source: Front Neurosci. 2022 Nov 8;16:1022308. doi: 10.3389/fnins.2022.1022308 (PMC9679532; doi:10.3389/fnins.2022.1022308)
Supplement: Supplementary file 1 [file Data_Sheet_1.PDF]

Supplementary Material

The following document is an export of the computational-notebook that was used to derive the probability density functions (PDFs). The computer algebra system (CAS) sympy was used for most of the process and all code as well as the results are included in this notebook. Most symbols used within the CAS match those used in the manuscript. An important exception is the IPD, which is denoted as φ instead of $\Delta\varphi$

1 SETUP

Load the necessary python package and define all symbols and functions.

```
from sympy import *
```

```
t = symbols('t', real=True)
X1 = Function('X1', real=True)(t)
X2 = Function('X2', real=True)(t)
Y1 = Function('Y1', real=True)(t)
Y2 = Function('Y2', real=True)(t)
X = Function('X', real=True)(t)
Y = Function('Y', real=True)(t)
r = symbols('r', real=True, postive=True)
ipd, psi = symbols('varphi, psi', real=True)
x, y = symbols('x, y', real=True)
sigma = symbols('sigma', real=True, positive=True)
Xi, Ups, xi, ups = symbols('Xi, Upsilon, xi, upsilon', real=True)
C = symbols('C', real=True, positive=True)
L = symbols('L', real=True)
p = symbols("p'", real=True, postive=True)
```

2 DERIVE JOINT IPD AND ILD PDF

2.1 Define the interaural Baseband

Define the general interaural baseband

```
N1 = (X1 + Y1*I)
N2 = (X2 + Y2*I)
Z1 = N1 / N2
Z1
```

$$\frac{X_1(t) + i\,Y_1(t)}{X_2(t) + i\,Y_2(t)}$$

Insert noise with tone

```
Z1 = Z1.subs(X1, X + C * cos(psi/2)).subs(X2, X + C * cos(-psi/2))
Z1 = Z1.subs(Y1, Y + C * sin(psi/2)).subs(Y2, Y + C * sin(-psi/2))
simplify(Z1)
```

$$\frac{C \cos\left(\frac{\psi}{2}\right) + i\left(C \sin\left(\frac{\psi}{2}\right) + Y(t)\right) + X(t)}{C \cos\left(\frac{\psi}{2}\right) - i\left(C \sin\left(\frac{\psi}{2}\right) - Y(t)\right) + X(t)}$$

This is Eq.(7) in the Manuscript

Extract real and imaginary part

```
Xi_eq_z1 = simplify(re(Z1))
Ups_eq_z1 = simplify(im(Z1))
Matrix([simplify(Xi_eq_z1), simplify(Ups_eq_z1)])
```

$$\left[\begin{matrix}\frac{-C^{2} \sin^{2}\left(\frac{\psi}{2}\right) + \left(C \cos\left(\frac{\psi}{2}\right) + X(t)\right)^{2} + Y^{2}(t)}{\left(C \sin\left(\frac{\psi}{2}\right) - Y(t)\right)^{2} + \left(C \cos\left(\frac{\psi}{2}\right) + X(t)\right)^{2}}\\ \frac{2C\left(C \cos\left(\frac{\psi}{2}\right) + X(t)\right) \sin\left(\frac{\psi}{2}\right)}{\left(C \sin\left(\frac{\psi}{2}\right) - Y(t)\right)^{2} + \left(C \cos\left(\frac{\psi}{2}\right) + X(t)\right)^{2}}\end{matrix}\right]$$

These are Eq.(8) and (9) in the Paper, for all further calculations, they are needed as functions of the instances x and y

```
xi_eq_z1 = Xi_eq_z1.subs(Y, y).subs(X, x)
ups_eq_z1 = Ups_eq_z1.subs(Y, y).subs(X, x)
xi_ups_vec_z1 = Matrix([xi_eq_z1, ups_eq_z1])
xi_ups_vec_z1
```

$$\left[\begin{matrix}\frac{-C^{2} \sin^{2}\left(\frac{\psi}{2}\right) + y^{2} + \left(C \cos\left(\frac{\psi}{2}\right) + x\right)^{2}}{\left(C \sin\left(\frac{\psi}{2}\right) - y\right)^{2} + \left(C \cos\left(\frac{\psi}{2}\right) + x\right)^{2}}\\ \frac{2C\left(C \cos\left(\frac{\psi}{2}\right) + x\right) \sin\left(\frac{\psi}{2}\right)}{\left(C \sin\left(\frac{\psi}{2}\right) - y\right)^{2} + \left(C \cos\left(\frac{\psi}{2}\right) + x\right)^{2}}\end{matrix}\right]$$

2.2 Derive PDF of Ξ and Υ

Solve aboves equation for x and y:

```
x_y_eq_z1 = solve(xi_ups_vec_z1 - Matrix([xi, ups]), Matrix([x, y]))
x_y_eq_z1 = Matrix(x_y_eq_z1[0])
x_y_eq_z1
```

$$\left[\begin{matrix}\frac{C\left(-\upsilon^{2} \cos\left(\frac{\psi}{2}\right) + 2\upsilon \sin\left(\frac{\psi}{2}\right) - \xi^{2} \cos\left(\frac{\psi}{2}\right) + 2\xi \cos\left(\frac{\psi}{2}\right) - \cos\left(\frac{\psi}{2}\right)\right)}{\upsilon^{2} + \xi^{2} - 2\xi + 1}\\ \frac{C\left(\upsilon^{2} + \xi^{2} - 1\right) \sin\left(\frac{\psi}{2}\right)}{\upsilon^{2} + \xi^{2} - 2\xi + 1}\end{matrix}\right]$$

some simplifications of $x(\xi, \upsilon)$

```
simpx = - C *(cos(psi/2) - (2*ups * sin(psi/2))/ ((xi - 1)**2 + ups**2))
if simpx.equals(x_y_eq_z1[0]): x_y_eq_z1[0] = simpx
x_y_eq_z1
```

$$\left[\begin{matrix}- C \left(- \frac{2 \upsilon \sin{\left(\frac{\psi}{2} \right)}}{\upsilon^{2} + \left(\xi - 1\right)^{2}} + \cos{\left(\frac{\psi}{2} \right)}\right)\\\frac{C \left(\upsilon^{2} + \xi^{2} - 1\right) \sin{\left(\frac{\psi}{2} \right)}}{\upsilon^{2} + \xi^{2} - 2 \xi + 1}\end{matrix}\right]$$

This is Eq.(12) in the Manuscript, next calculate the jacobian

```
eq_val = Matrix([xi, ups])
jac_z1 = x_y_eq_z1.jacobian(eq_val)
dxdy_z1 = simplify(det(jac_z1))
dxdy_z1
```

$$\frac{4 C^{2} \sin^{2}{\left(\frac{\psi}{2} \right)}}{\upsilon^{4} + 2 \upsilon^{2} \xi^{2} - 4 \upsilon^{2} \xi + 2 \upsilon^{2} + \xi^{4} - 4 \xi^{3} + 6 \xi^{2} - 4 \xi + 1}$$

some simplifications

```
simp_dxdy = - (2*C**2 * (cos(psi) - 1)) / ((xi - 1)**2 + ups**2)**2
if dxdy_z1.equals(simp_dxdy): dxdy_z1 = simp_dxdy
dxdy_z1
```

$$- \frac{2 C^{2} \left(\cos{\left(\psi \right)} - 1\right)}{\left(\upsilon^{2} + \left(\xi - 1\right)^{2}\right)^{2}}$$

Start with a bivariant normal distribution

```
norm_pdf = 1 / (2 * pi * sigma**2) * exp(-(x**2 + y**2) / (2 * sigma**2))
norm_pdf
```

$$\frac{e^{\frac{- x^{2} - y^{2}}{2 \sigma^{2}}}}{2 \pi \sigma^{2}}$$

And replace the variables

```
xiups_pdf_z1 = (norm_pdf * dxdy_z1).subs(x, x_y_eq_z1[0]).subs(y, x_y_eq_z1[1])
xiups_pdf_z1
```

$$- \frac{C^{2} \left(\cos{\left(\psi \right)} - 1\right) e^{\frac{- C^{2} \left(- \frac{2 \upsilon \sin{\left(\frac{\psi}{2} \right)}}{\upsilon^{2} + \left(\xi - 1\right)^{2}} + \cos{\left(\frac{\psi}{2} \right)}\right)^{2} - \frac{C^{2} \left(\upsilon^{2} + \xi^{2} - 1\right)^{2} \sin^{2}{\left(\frac{\psi}{2} \right)}}{\left(\upsilon^{2} + \xi^{2} - 2 \xi + 1\right)^{2}}}{2 \sigma^{2}}}}{\pi \sigma^{2} \left(\upsilon^{2} + \left(\xi - 1\right)^{2}\right)^{2}}$$

Some Simplifications:

```
exp_term = xiups_pdf_z1.args[-1].args[0]
simp_exp = (-(C**2 * (xi**2 + ups**2 - 2 * xi * cos(psi)
                    - 2 * ups * sin(psi) + 1))
            / (2 * sigma**2 *((xi - 1)**2 + ups**2)))
if simp_exp.equals(exp_term): xiups_pdf_z1 = xiups_pdf_z1.subs(exp_term, simp_exp)
xiups_pdf_z1
```

$$-\frac{C^{2}\left(\cos\left(\psi\right)-1\right)e^{-\frac{C^{2}\left(\upsilon^{2}-2\upsilon\sin\left(\psi\right)+\xi^{2}-2\xi\cos\left(\psi\right)+1\right)}{2\sigma^{2}\left(\upsilon^{2}+\left(\xi-1\right)^{2}\right)}}}{\pi\sigma^{2}\left(\upsilon^{2}+\left(\xi-1\right)^{2}\right)^{2}}$$

This is Eq. (13) of the Manuscript

2.3 Convert into polar coordinates to gain the PDF of IAR and IPD

```
xi_polar = r * cos(ipd)
ups_polar = r * sin(ipd)
```

```
ripd_pdf = (xiups_pdf_z1.subs(xi, xi_polar).subs(ups, ups_polar) * r)
ripd_pdf
```

$$-\frac{C^{2}r\left(\cos\left(\psi\right)-1\right)e^{-\frac{C^{2}\left(r^{2}\sin^{2}\left(\varphi\right)+r^{2}\cos^{2}\left(\varphi\right)-2r\sin\left(\psi\right)\sin\left(\varphi\right)-2r\cos\left(\psi\right)\cos\left(\varphi\right)+1\right)}{2\sigma^{2}\left(r^{2}\sin^{2}\left(\varphi\right)+\left(r\cos\left(\varphi\right)-1\right)^{2}\right)}}}{\pi\sigma^{2}\left(r^{2}\sin^{2}\left(\varphi\right)+\left(r\cos\left(\varphi\right)-1\right)^{2}\right)^{2}}$$

2.3.1 Simplify the final equation

```
exp_term = ripd_pdf.args[-1].args[0]
simp_term = (-(C**2 * (r**2 - 2 * r * cos(ipd - psi) + 1))
             / (2 * sigma**2 * (r**2 - 2 * r * cos(ipd) + 1)))
if simp_term.equals(exp_term): ripd_pdf = ripd_pdf.subs(exp_term, simp_term)
ripd_pdf
```

$$-\frac{C^{2}r\left(\cos\left(\psi\right)-1\right)e^{-\frac{C^{2}\left(r^{2}-2r\cos\left(\psi-\varphi\right)+1\right)}{2\sigma^{2}\left(r^{2}-2r\cos\left(\varphi\right)+1\right)}}}{\pi\sigma^{2}\left(r^{2}\sin^{2}\left(\varphi\right)+\left(r\cos\left(\varphi\right)-1\right)^{2}\right)^{2}}$$

```
term = ripd_pdf.args[-3]
simp_term = 1 / (r**2 - 2*r*cos(ipd) + 1)**2
if simp_term.equals(term): ripd_pdf = ripd_pdf.subs(term, simp_term)
ripd_pdf
```

$$-\frac{C^{2}r\left(\cos\left(\psi\right)-1\right)e^{-\frac{C^{2}\left(r^{2}-2r\cos\left(\psi-\varphi\right)+1\right)}{2\sigma^{2}\left(r^{2}-2r\cos\left(\varphi\right)+1\right)}}}{\pi\sigma^{2}\left(r^{2}-2r\cos\left(\varphi\right)+1\right)^{2}}$$

this is Eq. (14) of the Manuscript

2.3.2 Derive the transform from IAR to ILD

```
r_rep = 10**(L/20)
dr = diff(r_rep, L)
simplify(dr)
```

$$\frac{10^{\frac{L}{20}}\log\left(10\right)}{20}$$

3 DERIVE JOINT IPD AND P' PDF

3.1 Define the interaural Baseband

Define the general interaural baseband

```
Z2 = (expand((X1 + Y1*I) * (X2 + Y2*I).conjugate()))
Z2
```

$$\mathrm{X}_1(t)\,\mathrm{X}_2(t) - i\,\mathrm{X}_1(t)\,\mathrm{Y}_2(t) + i\,\mathrm{X}_2(t)\,\mathrm{Y}_1(t) + \mathrm{Y}_1(t)\,\mathrm{Y}_2(t)$$

Insert noise with tone

```
Z2 = (Z2.subs(X1, X + C * cos(psi/2)).subs(X2, X + C * cos(-psi/2)))
Z2 = (Z2.subs(Y1, Y + C * sin(psi/2)).subs(Y2, Y + C * sin(-psi/2)))
Z2 = expand(Z2)
Z2
```

$$-C^2\sin^2\left(\frac{\psi}{2}\right) + 2iC^2\sin\left(\frac{\psi}{2}\right)\cos\left(\frac{\psi}{2}\right) + C^2\cos^2\left(\frac{\psi}{2}\right) + 2iCX(t)\sin\left(\frac{\psi}{2}\right) + 2CX(t)\cos\left(\frac{\psi}{2}\right) + X^2(t) + Y^2(t)$$

Extract real and imaginary part

```
Xi_eq_z2 = simplify(re(Z2))
Ups_eq_z2 = simplify(im(Z2))
Matrix([Xi_eq_z2, Ups_eq_z2])
```

$$\begin{bmatrix} -2C^2\sin^2\left(\frac{\psi}{2}\right) + C^2 + 2CX(t)\cos\left(\frac{\psi}{2}\right) + X^2(t) + Y^2(t) \\ 2C\left(\frac{C\sin(\psi)}{2} + X(t)\sin\left(\frac{\psi}{2}\right)\right) \end{bmatrix}$$

```
ups_eq_z2 = Ups_eq_z2.subs(Y, y).subs(X, x)
xi_eq_z2 = Xi_eq_z2.subs(Y, y).subs(X, x)
```

3.2 Derive PDF of Ξ and Υ

Solve aboves equation for x and y:

```
x_eq_z2 = simplify(solve(ups_eq_z2 - ups, x)[0])
y_eq_z2 = simplify(solve(xi_eq_z2.subs(x, x_eq_z2) - xi, y)[0])
Matrix([x_eq_z2, y_eq_z2])
```

$$\begin{bmatrix} \frac{-C^2\sin(\psi)+\upsilon}{2C\sin\left(\frac{\psi}{2}\right)} \\ -\frac{\sqrt{8C^4\sin^4\left(\frac{\psi}{2}\right) - 4C^4\sin^2\left(\frac{\psi}{2}\right) + C^4\sin^2(\psi) + 4C^2\xi\sin^2\left(\frac{\psi}{2}\right) - \upsilon^2}}{2C\sin\left(\frac{\psi}{2}\right)} \end{bmatrix}$$

```
eq_matrix = Matrix([x_eq_z2, y_eq_z2])
eq_val = Matrix([xi, ups])
jac_z2 = eq_matrix.jacobian(eq_val)
dxdy_z2 = simplify(det(jac_z2))
```

```
dxdy_z2
```

$$\frac{1}{2\sqrt{8C^4\sin^4\left(\frac{\psi}{2}\right) - 4C^4\sin^2\left(\frac{\psi}{2}\right) + C^4\sin^2(\psi) + 4C^2\xi\sin^2\left(\frac{\psi}{2}\right) - \upsilon^2}}$$

Start with a bivariant normal distribution

```
norm_pdf = 1 / (2 * pi * sigma**2) * exp(-(x**2 + y**2) / (2 * sigma**2))
norm_pdf
```

$$\frac{e^{\frac{-x^2-y^2}{2\sigma^2}}}{2\pi\sigma^2}$$

```
xiups_pdf_z2 = (norm_pdf * dxdy_z2).subs(x, x_eq_z2).subs(y, y_eq_z2)
xiups_pdf_z2
```

$$\frac{e^{\frac{-\frac{\left(-C^2\sin(\psi)+\upsilon\right)^2}{4C^2\sin^2\left(\frac{\psi}{2}\right)} - \frac{8C^4\sin^4\left(\frac{\psi}{2}\right)-4C^4\sin^2\left(\frac{\psi}{2}\right)+C^4\sin^2(\psi)+4C^2\xi\sin^2\left(\frac{\psi}{2}\right)-\upsilon^2}{4C^2\sin^2\left(\frac{\psi}{2}\right)}}{2\sigma^2}}}{4\pi\sigma^2\sqrt{8C^4\sin^4\left(\frac{\psi}{2}\right) - 4C^4\sin^2\left(\frac{\psi}{2}\right) + C^4\sin^2(\psi) + 4C^2\xi\sin^2\left(\frac{\psi}{2}\right) - \upsilon^2}}$$

3.3 Convert into polar coordinates to gain the PDF of P' and IPD

```
xi_polar = p * cos(ipd)
ups_polar = p * sin(ipd)
```

```
pipd_pdf = 2 * xiups_pdf_z2.subs(xi, xi_polar).subs(ups, ups_polar) * p
pipd_pdf
```

$$\frac{p' e^{\frac{-\frac{\left(-C^2\sin(\psi)+p'\sin(\varphi)\right)^2}{4C^2\sin^2\left(\frac{\psi}{2}\right)} - \frac{8C^4\sin^4\left(\frac{\psi}{2}\right)-4C^4\sin^2\left(\frac{\psi}{2}\right)+C^4\sin^2(\psi)+4C^2p'\sin^2\left(\frac{\psi}{2}\right)\cos(\varphi)-p'^2\sin^2(\varphi)}{4C^2\sin^2\left(\frac{\psi}{2}\right)}}{2\sigma^2}}}{2\pi\sigma^2\sqrt{8C^4\sin^4\left(\frac{\psi}{2}\right) - 4C^4\sin^2\left(\frac{\psi}{2}\right) + C^4\sin^2(\psi) + 4C^2p'\sin^2\left(\frac{\psi}{2}\right)\cos(\varphi) - p'^2\sin^2(\varphi)}}$$

3.3.1 Simplify the PDF

Simplify the term under the square root:

```
sqrt_term =pipd_pdf.args[4].args[0]
simp_term = 2*C**2 * sin(psi/2)**2 * (2 * p * cos(ipd)
                                    - C**2 * (cos(psi) -1))
                                    - p**2 * sin(ipd)**2
if simp_term.equals(sqrt_term): pipd_pdf = pipd_pdf.subs(sqrt_term, simp_term)
pipd_pdf
```

$$\frac{p' e^{\frac{-\frac{\left(-C^{2}\sin\left(\psi\right)+p'\sin\left(\varphi\right)\right)^{2}}{4C^{2}\sin^{2}\left(\frac{\psi}{2}\right)}-\frac{2C^{2}\left(-C^{2}\left(\cos\left(\psi\right)-1\right)+2p'\cos\left(\varphi\right)\right)\sin^{2}\left(\frac{\psi}{2}\right)-p'^{2}\sin^{2}\left(\varphi\right)}{4C^{2}\sin^{2}\left(\frac{\psi}{2}\right)}}{2\sigma^{2}}}}{2\pi\sigma^{2}\sqrt{2C^{2}\left(-C^{2}\left(\cos\left(\psi\right)-1\right)+2p'\cos\left(\varphi\right)\right)\sin^{2}\left(\frac{\psi}{2}\right)-p'^{2}\sin^{2}\left(\varphi\right)}}$$

Simplify the exponential term:

```
exp_term = pipd_pdf.args[5].args[0]
mult_term = exp_term.func(*exp_term.args[0:2])
simp_exp_term = (exp_term.args[2].args[0] * mult_term
                 + exp_term.args[2].args[1] * mult_term)
if exp_term.equals(simp_exp_term):
  pipd_pdf = pipd_pdf.subs(exp_term , simp_exp_term)
pipd_pdf
```

$$\frac{p' e^{-\frac{\left(-C^{2}\sin\left(\psi\right)+p'\sin\left(\varphi\right)\right)^{2}}{8C^{2}\sigma^{2}\sin^{2}\left(\frac{\psi}{2}\right)}-\frac{2C^{2}\left(-C^{2}\left(\cos\left(\psi\right)-1\right)+2p'\cos\left(\varphi\right)\right)\sin^{2}\left(\frac{\psi}{2}\right)-p'^{2}\sin^{2}\left(\varphi\right)}{8C^{2}\sigma^{2}\sin^{2}\left(\frac{\psi}{2}\right)}}}{2\pi\sigma^{2}\sqrt{2C^{2}\left(-C^{2}\left(\cos\left(\psi\right)-1\right)+2p'\cos\left(\varphi\right)\right)\sin^{2}\left(\frac{\psi}{2}\right)-p'^{2}\sin^{2}\left(\varphi\right)}}$$

Start with the first Part of the exponential and split it into those with with and with out the r term

```
term = pipd_pdf.args[5].args[0]
eterm = expand(term)
```

```
non_p = eterm.func(*[t for t in eterm.args if p not in t.free_symbols])
non_p = simplify(non_p)
non_p
```

$$-\frac{C^{2}}{2\sigma^{2}}$$

```
with_p = eterm.func(*[t for t in eterm.args if p in t.free_symbols])
with_p = simplify(with_p)
with_p
```

$$\frac{p'\left(\cos\left(\varphi\right)-\cos\left(\psi-\varphi\right)\right)}{2\sigma^{2}\left(\cos\left(\psi\right)-1\right)}$$

```
pipd_pdf = pipd_pdf.subs(term, non_p + with_p)
pipd_pdf
```

$$\frac{p' e^{-\frac{C^{2}}{2\sigma^{2}}+\frac{p'\left(\cos\left(\varphi\right)-\cos\left(\psi-\varphi\right)\right)}{2\sigma^{2}\left(\cos\left(\psi\right)-1\right)}}}{2\pi\sigma^{2}\sqrt{2C^{2}\left(-C^{2}\left(\cos\left(\psi\right)-1\right)+2p'\cos\left(\varphi\right)\right)\sin^{2}\left(\frac{\psi}{2}\right)-p'^{2}\sin^{2}\left(\varphi\right)}}$$

3.4 Find the integration boundaries:

```
rel = sqrt_term
p_limits = solve(rel, p)
```

```
limits = p_limits[1]
limits
```

$$\frac{C^{2}\left(\sqrt{4\sin^{4}\left(\frac{\psi}{2}\right)\sin^{2}\left(\varphi\right)+4\sin^{4}\left(\frac{\psi}{2}\right)-4\sin^{2}\left(\frac{\psi}{2}\right)\sin^{2}\left(\varphi\right)+\sin^{2}\left(\psi\right)\sin^{2}\left(\varphi\right)}+2\sin^{2}\left(\frac{\psi}{2}\right)\cos\left(\varphi\right)\right)}{\sin^{2}\left(\varphi\right)}$$

The term under the square root can be simplified by quite a bit:

```
simp_eq = 4*sin(psi/2)**4
limits.args[2].args[0].args[0].equals(simp_eq)
```

```
True
```

resulting in the limit:

```
limit = simplify(limits.subs(limits.args[2].args[0].args[0], simp_eq))
limit
```

$$\frac{2C^{2}\left(\cos\left(\varphi\right)+1\right)\sin^{2}\left(\frac{\psi}{2}\right)}{\sin^{2}\left(\varphi\right)}$$

```
simp_limit = (C**2*(cos(psi)-1)) / (cos(ipd)-1)
limit = simp_limit if limit.equals(simp_limit) else limit
limit
```

$$\frac{C^{2}\left(\cos\left(\psi\right)-1\right)}{\cos\left(\varphi\right)-1}$$

Inverse limit

```
Matrix(solve(limit - p, ipd))[1]
```

$$\operatorname{acos}\left(\frac{C^{2}\cos\left(\psi\right)-C^{2}+p'}{p'}\right)$$
